# Differences in clinical characteristics of cervical spine injuries in older adults by external causes: a multicenter study of 1512 cases

**DOI:** 10.1038/s41598-022-19789-y

**Published:** 2022-09-23

**Authors:** Noriaki Yokogawa, Satoshi Kato, Takeshi Sasagawa, Hiroyuki Hayashi, Hiroyuki Tsuchiya, Kei Ando, Hiroaki Nakashima, Naoki Segi, Toru Funayama, Fumihiko Eto, Akihiro Yamaji, Satoshi Nori, Junichi Yamane, Takeo Furuya, Atsushi Yunde, Hideaki Nakajima, Tomohiro Yamada, Tomohiko Hasegawa, Yoshinori Terashima, Ryosuke Hirota, Hidenori Suzuki, Yasuaki Imajo, Shota Ikegami, Masashi Uehara, Hitoshi Tonomura, Munehiro Sakata, Ko Hashimoto, Yoshito Onoda, Kenichi Kawaguchi, Yohei Haruta, Nobuyuki Suzuki, Kenji Kato, Hiroshi Uei, Hirokatsu Sawada, Kazuo Nakanishi, Kosuke Misaki, Hidetomi Terai, Koji Tamai, Eiki Shirasawa, Gen Inoue, Kenichiro Kakutani, Yuji Kakiuchi, Katsuhito Kiyasu, Hiroyuki Tominaga, Hiroto Tokumoto, Yoichi Iizuka, Eiji Takasawa, Koji Akeda, Norihiko Takegami, Haruki Funao, Yasushi Oshima, Takashi Kaito, Daisuke Sakai, Toshitaka Yoshii, Tetsuro Ohba, Bungo Otsuki, Shoji Seki, Masashi Miyazaki, Masayuki Ishihara, Seiji Okada, Shiro Imagama, Kota Watanabe

**Affiliations:** 1 Department of Orthopaedic Surgery, Graduate School of Medical Sciences, Kanazawa University, 13-1 Takara-machi, Kanazawa, Ishikawa 920-8641 Japan; 2 Department of Orthopedics Surgery, Toyama Prefectural Central Hospital, Toyama, Japan; 3 Department of Orthopaedic Surgery, Tonami General Hospital, Tonami, Japan; 4 Department of Orthopedic Surgery, Nagoya University, Graduate School of Medicine, Nagoya, Japan; 5 Department of Orthopaedic Surgery, Faculty of Medicine, University of Tsukuba, Ibaraki, Japan; 6 Department of Orthopaedic Surgery, Graduate School of Comprehensive Human Sciences, University of Tsukuba, Ibaraki, Japan; 7 Department of Orthopaedic Surgery, Ibaraki Seinan Medical Center Hospital, Ibaraki, Japan; 8 Department of Orthopaedic Surgery, Keio University School of Medicine, Tokyo, Japan; 9 Department of Orthopaedic Surgery, National Hospital Organization Murayama Medical Center, Tokyo, Japan; 10 Department of Orthopaedic Surgery, Graduate School of Medicine, Chiba University, Chiba, Japan; 11 Department of Orthopaedics and Rehabilitation Medicine, Faculty of Medical Sciences, University of Fukui, Fukui, Japan; 12 Department of Orthopaedic Surgery, Hamamatsu University School of Medicine, Shizuoka, Japan; 13 Department of Orthopaedic Surgery, Nagoya Kyoritsu Hospital, Nagoya, Japan; 14 Department of Orthopaedic Surgery, Sapporo Medical University, Sapporo, Japan; 15 Department of Orthopaedic Surgery, Matsuda Orthopedic Memorial Hospital, Sapporo, Japan; 16 Department of Orthopaedic Surgery, Yamaguchi University Graduate School of Medicine, Yamaguchi, Japan; 17 Department of Orthopaedic Surgery, Shinshu University School of Medicine, Nagano, Japan; 18 Department of Orthopaedics, Graduate School of Medical Science, Kyoto Prefectural University of Medicine, Kyoto, Japan; 19 Department of Orthopaedics, Saiseikai Shiga Hospital, Ritto, Shiga Japan; 20 Department of Orthopaedic Surgery, Tohoku University Graduate School of Medicine, Sendai, Miyagi Japan; 21 Department of Orthopaedic Surgery, Graduate School of Medical Sciences, Kyushu University, Fukuoka, Japan; 22 Department of Orthopaedic Surgery, Nagoya City University Graduate School of Medical Sciences, Nagoya, Japan; 23 Department of Orthopaedic Surgery, Nihon University Hospital, Tokyo, Japan; 24 Department of Orthopaedic Surgery, Nihon University School of Medicine, Tokyo, Japan; 25 Department of Orthopedics, Traumatology and Spine Surgery, Kawasaki Medical School, Okayama, Japan; 26 Department of Orthopaedic Surgery, Osaka City University Graduate School of Medicine, Osaka, Japan; 27 Department of Orthopaedic Surgery, Kitasato University School of Medicine, Sagamihara, Kanagawa Japan; 28 Department of Orthopaedic Surgery, Kobe University Graduate School of Medicine, Kobe, Japan; 29 Department of Orthopaedic Surgery, Kochi Medical School, Kochi University, Nankoku, Japan; 30 Department of Orthopaedic Surgery, Graduate School of Medical and Dental Sciences, Kagoshima University, Kagoshima, Japan; 31 Department of Orthopaedic Surgery, Gunma University, Graduate School of Medicine, Maebashi, Gunma Japan; 32 Department of Orthopaedic Surgery, Mie University Graduate School of Medicine, Tsu, Mie Japan; 33 Department of Orthopaedic Surgery, School of Medicine, International University of Health and Welfare, Chiba, Japan; 34 Department of Orthopaedic Surgery, International University of Health and Welfare Narita Hospital, Chiba, Japan; 35 Department of Orthopaedic Surgery and Spine and Spinal Cord Center, International University of Health and Welfare Mita Hospital, Tokyo, Japan; 36 Department of Orthopaedic Surgery, The University of Tokyo Hospital, Tokyo, Japan; 37 Department of Orthopaedic Surgery, Osaka University Graduate School of Medicine, Osaka, Japan; 38 Department of Orthopedics Surgery, Surgical Science, Tokai University School of Medicine, Kanagawa, Japan; 39 Department of Orthopaedic Surgery, Tokyo Medical and Dental University, Tokyo, Japan; 40 Department of Orthopaedic Surgery, University of Yamanashi, Kofu, Yamanashi Japan; 41 Department of Orthopaedic Surgery, Graduate School of Medicine, Kyoto University, Kyoto, Japan; 42 Department of Orthopaedic Surgery, Faculty of Medicine, University of Toyama, Toyama, Japan; 43 Department of Orthopaedic Surgery, Faculty of Medicine, Oita University, Oita, Japan; 44 Department of Orthopaedic Surgery, Kansai Medical University Hospital, Osaka, Japan

**Keywords:** Medical research, Geriatrics, Trauma

## Abstract

Although traumatic cervical spine injuries in older adults are commonly caused by minor traumas, such as ground-level falls, their prognosis is often unfavorable. Studies examining the clinical characteristics of cervical spine injuries in older adults according to the external cause of injury are lacking. This study included 1512 patients of ≥ 65 years of age with traumatic cervical spine injuries registered in a Japanese nationwide multicenter database. The relationship between the external causes and clinical characteristics, as well as factors causing unfavorable outcomes at the ground-level falls, were retrospectively reviewed and examined. When fall-induced cervical spine injuries were categorized and compared based on fall height, the patients’ backgrounds and injury statuses differed significantly. Of note, patients injured from ground-level falls tended to have poorer pre-injury health conditions, such as medical comorbidities and frailty, compared with those who fell from higher heights. For ground-level falls, the mortality, walking independence, and home-discharge rates at 6 months post-injury were 9%, 67%, and 80%, respectively, with preexisting medical comorbidities and frailty associated with unfavorable outcomes, independent of age or severity of neurological impairment at the time of injury.

## Introduction

The global percentage of individuals aged 65 years and over increased from 6% in 1990 to 9% in 2019 and is projected to rise further to 16% by 2050, with one in six people worldwide aged ≥ 65 years^1^. Japan has the highest aging rate worldwide, with 29% of the population aged ≥ 65 years in 2021, which is approximately one-third of the entire Japanese population^2^.

The number of traumatic injuries in older people has also been rising, and injuries due to falls are a serious public health concern in aging societies^3,4^. Approximately 30% of community-dwelling adults aged 65 years and older fall at least once a year, with the frequency of falls increasing with age^5,6^. Approximately 10% of all falls result in serious injuries among older people, and cervical spine injury is one of the most severe injuries with high morbidity and mortality rates^7,8^. Fall-induced cervical spine injuries are likely to occur even with relatively minor trauma in older people, and the incidence is increasing in developed countries^9–12^.

The World Health Organization categorizes falls into four patterns: (1) falls from ground-level, (2) falls from heights of less than one meter, (3) falls from heights of one meter or more, and (4) being struck or crushed by a falling object^13^. This categorization suggests that clinical characteristics may vary depending on the fall height, even for the same fall-induced injury. There have been a few reports addressing the differences in the clinical characteristics of spinal cord injuries depending on the fall height^14,15^; however, there is a lack of studies on the clinical characteristics, including prognoses, in cohorts of older patients with traumatic cervical spine injury based on the external cause of injury with a focus on the fall height. Furthermore, the causes of poor prognoses with minor injuries in older people, such as due to a fall from ground level, have not been clarified.

In this study, a nationwide survey of cervical spine injuries in Japan's hyper-aging population was conducted, with the aim of answering the following questions: (1) To what extent do clinical characteristics and treatment outcomes differ by the external cause of injury, particularly by fall height? and (2) What are the unfavorable prognostic factors in cases of ground-level falls, the most common form of trauma? This nationwide survey would provide important evidence to prevent cervical spine injuries and improve their prognosis of in older adults in a rapidly aging society.

## Methods

### Study design

Data from patients of ≥ 65 years of age at the time of injury who had traumatic cervical spine injuries between 2010 and 2020 and had been registered in the multicenter database of the Japan Association of Spine Surgeons with Ambition were retrospectively analyzed. Cervical spine injuries included cervical fractures, dislocations, spinal cord injuries, and the combinations thereof. The criteria for enrollment in the database were patients who required in-hospital treatment and had at least 3 months of follow-up. Patient data were collected from medical records and imaging data. The protocol of this study was approved by the Ethics Committee of Kanazawa University Hospital, an institutional review board of the representative facility in this multicenter study (no. 3352-1). Informed consent was obtained in the form of an opt-out procedure at each institute, and patients who declined to participate were excluded. This study was carried out in accordance with relevant guidelines and regulations.

### Variables, classifications, and outcomes

Variables included the external cause of injury, pre-injury patient demographics (age, sex, body mass index, residence status, walking ability, medical comorbidities, Charlson Comorbidity Index [CCI]^16^, number of medications, modified 5-item frailty index [mFI-5], and presence of cervical ligamentous ossification), blood tests on admission (total protein and hemoglobin), injury status (presence of cervical fracture/dislocation, neurological impairment, and associated injury), and treatment status (steroid administration and surgery). The mFI-5 is a surrogate index for frailty, which is calculated based on the number of applicable items in the five categories of activities of daily living. These include requiring assistance and the presence of hypertension, diabetes, congestive heart failure, and chronic obstructive pulmonary disease^17,18^. Neurological impairment was assessed using the American Spinal Injury Association Impairment Scale (AIS)^19^. External causes of injury were classified as falling from the ground level, falling from heights below 1 m (low fall), falling from heights above 1 m (high fall), traffic accidents, and others (including those with unspecified reasons). In addition, external causes were examined by age, and patients were divided into three age groups as follows: 65–75 years, 75–85 years, and ≥ 85 years of age. The outcomes of interest were mortality, complication rate (respiratory impairment, dysphagia, deep venous thrombosis, pulmonary embolism, pneumonia, urinary tract infection, cerebral infarction, myocardial infarction, and delirium), walking independence rate, and home-discharge rate (returned home, admitted to a facility, or in hospital including rehabilitation hospital) at 6 months post-injury. Six months of follow-up data were available for 79% of the cases. The home-discharge rate was applicable for patients who lived at home before the injury, and the walking independence rate was applicable for patients who could walk independently before the injury. Independent walking was defined as the possibility of walking with or without a cane without any assistance from a physiotherapist.

### Statistical analysis

The differences in each parameter and outcome based on the fall height were analyzed using the Cochran–Armitage test or Jonckheere–Terpstra test for trend. For patients injured from ground-level falls, multivariate analyses were performed using forward stepwise logistic regression (likelihood ratio) to identify the prognostic factors for unfavorable outcomes, including mortality, walking disability, and difficulty in home discharge. The explanatory variables were the following key parameters that have been reported to impact the prognosis of cervical spine injuries^20–24^: age at injury, sex, preexisting medical comorbidities (CCI), frailty (mFI-5), presence of cervical fracture/dislocation, neurological impairment upon injury (AIS; E:0, D:1, C:2, B:3, A:4), and the presence of an associated injury. The variables were entered in blocks according to the dimensions of each explanatory variable. The P-value was set to < 0.05. Statistical analyses were performed using SPSS software (Version 23; IBM Corp., Armonk, NY, USA) and JMP software (Version 16.1.0; SAS Institute, Cary, NC, USA).

## Results

### External causes of injury

**Figure 1:**
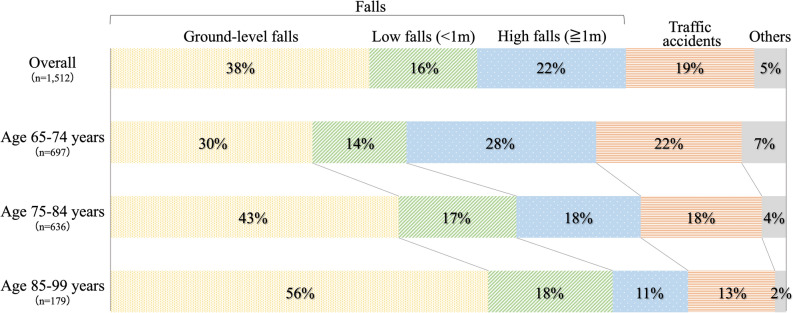
External causes of injury by age group.

In total, 1512 patients with traumatic cervical spine injuries were identified in our database during the study period from 78 institutions throughout Japan. The most common cause of injury was ground-level falls (38%), followed by high falls (22%), traffic accidents (19%), low falls (16%), and others (5%); thus, three-quarters of all injuries were caused by falls. The proportion of ground-level falls increased with age, and more than half of the patients aged 85 years and older were injured by falls from ground level (Fig. 1).

### Patient characteristics by the external cause of injury

The mean age of the patients studied was approximately 75.8 years, and on average, patients who were injured from a fall were relatively older than others. Two-thirds of the total number of patients were men, with a higher proportion of women in patients of injuries due to traffic accidents than in others. More than 95% of patients were living at home and walking independently before the injury. Overall, most patients had some underlying health condition, and the prevalence of each condition was relatively higher in patients who had fallen, with a mean CCI > 4. The mean mFI-5 was slightly lower in the patients injured during traffic accidents. Blood test data at the time of admission, including mean serum total protein and hemoglobin, showed little difference according to the cause of injury. A radiological examination showed that 22% of patients had ossification of the posterior longitudinal ligament (OPLL), and 13% had diffuse idiopathic skeletal hyperostosis (DISH) in the cervical spine. These rates were relatively higher in patients who were injured by falls than in others. Cervical bony injuries (fractures and/or dislocations) were found in 59% of the patients, of which half had associated spinal cord injuries, and 41% had cervical spinal cord injuries without bony injuries. In patients who were injured in traffic accidents, cervical bony injuries were frequently observed; in contrast, patients who were injured from falling were likely to sustain cervical spinal cord injury without bony injuries, with the majority showing AIS grade C or D. Associated injuries were observed in a quarter of the patients, and head trauma was relatively common. Multiple injuries were more frequent with traffic accidents. Regarding treatment, surgery was performed in approximately 60% of patients, both those injured during falls and those injured in traffic accidents (Table 1, Supplementary Table 1).

**Table 1:** Patient characteristics by the external cause of injury.

	Overall (n = 1512)	Falls (n = 1153)	Traffic accidents (n = 287)	Others (including unspecified) (n = 72)
**Pre-injury patient demographics**				
Mean age at injury (years)	75.8	76.2	74.8	73.2
Sex: men, n (%)	1007 (66.6)	782 (67.8)	166 (57.8)	59 (81.9)
Mean body mass index (kg/m^2^)	22.1	22.1	22.1	22.3
Residence status: home, n (%)	1439 (97.0)	1093 (96.3)	276 (99.3)	70 (100)
Walking capacity: walking w/ or w/o a cane, n (%)	1431 (95.5)	1084 (94.8)	281 (98.6)	66 (94.3)
Mean Charlson comorbidity index	0.8	0.8	0.6	0.7
Mean number of medications	3.9	4.1	3.0	3.9
Mean modified 5-item frailty index	0.8	0.8	0.7	0.8
**Presence of cervical ligamentous ossification**				
OPLL, n (%)	332 (22.0)	270 (23.4)	45 (15.7)	17 (23.6)
OLF, n (%)	25 (1.7)	21 (1.8)	4 (1.4)	0 (0)
DISH, n (%)	189 (12.5)	156 (13.6)	24 (8.4)	9 (12.5)
**Injury status**				
Cervical vertebral fracture, n (%)	834 (55.2)	595 (51.6)	198 (69.0)	41 (56.9)
Cervical dislocation, n (%)	229 (15.2)	162 (14.1)	59 (20.6)	8 (11.1)
Spinal cord injury, n (%)	1056 (69.8)	835 (72.4)	171 (59.6)	50 (69.4)
w/ bony injury, n (%)	441 (29.2)	321 (27.8)	97 (33.8)	23 (31.9)
w/o bony injury, n (%)	615 (40.7)	514 (44.6)	74 (25.8)	27 (37.5)
ASIA impairment scale				
A, n (%)	125 (8.3)	96 (8.4)	23 (8.0)	6 (8.3)
B, n (%)	68 (4.5)	57 (5.0)	8 (2.8)	3 (4.2)
C, n (%)	336 (22.3)	266 (23.2)	52 (18.2)	18 (25.0)
D, n (%)	519 (34.5)	409 (35.7)	87 (30.4)	23 (31.9)
Associated injuries, n (%)	411 (27.2)	269 (23.3)	134 (46.7)	8 (11.1)
**Treatment status**				
Surgery implementation, n (%)	903 (59.7)	676 (58.6)	174 (60.6)	53 (73.6)

Records with unknown or missing values were excluded from the analysis.

*OPLL* Ossification of the posterior longitudinal ligament, *OLF* Ossification of the ligamentum flavum, *DISH* Diffuse idiopathic skeletal hyperostosis, *ASIA* American Spinal Injury Association.

### Patient outcomes at 6 months post-injury by the external cause of injury

The overall mortality rate was 7%, with a rate of 8% for patients in fall accidents and 7% for those in traffic accidents. The overall complication rate was 36%, with relatively high incidences of respiratory impairment, dysphagia, pneumonia, urinary tract infection, and delirium. The overall walking independence rate and home-discharge rate were 73% and 82%, respectively, and 14% of the patients were still hospitalized after 6 months (Table 2).

**Table 2:** Patient outcomes at 6 months post-injury by the external cause of injury.

	Overall	Falls	Traffic accidents	Others (including unspecified)
**Mortality and complication rate**				
Number of subjects^a^	1198	909	227	62
Mortality, n (%)	89 (7.4)	70 (7.7)	16 (7.0)	3 (4.8)
Complications, n (%)	423 (36.4)	328 (37.2)	74 (34.1)	21 (33.9)
Respiratory impairment, n (%)	81 (6.8)	61 (6.8)	16 (7.1)	4 (6.5)
Dysphagia, n (%)	81 (6.8)	64 (7.1)	14 (6.2)	3 (4.8)
Deep venous thrombosis, n (%)	20 (1.7)	14 (1.6)	5 (2.2)	1 (1.6)
Pulmonary embolism, n (%)	6 (0.5)	6 (0.7)	0 (0)	0 (0)
Pneumonia, n (%)	115 (9.7)	93 (10.3)	16 (7.1)	6 (9.7)
Urinary tract infection, n (%)	110 (9.2)	83 (9.2)	17 (7.5)	10 (16.1)
Cerebral infarction, n (%)	13 (1.1)	10 (1.1)	3 (1.3)	0 (0)
Myocardial infarction, n (%)	2 (0.2)	2 (0.2)	0 (0)	0 (0)
Delirium, n (%)	95 (8.0)	72 (8.0)	19 (8.4)	4 (6.5)
**Walking independence rate**				
Number of subjects^b^	1057	795	206	56
Walking capacity: walking w/ or w/o a cane, n (%)	768 (72.7)	572 (71.9)	155 (75.2)	41 (73.2)
**Home-discharge rate**				
Number of subjects^c^	1,061	801	203	57
Returned to home, n (%)	857 (81.5)	650 (81.1)	159 (78.3)	48 (84.2)
Admitted to a facility, n (%)	61 (5.8)	48 (6.0)	11 (5.4)	2 (3.5)
In hospital, n (%)	143 (13.6)	103 (12.9)	33 (16.2)	7 (12.3)

Records with unknown or missing values were excluded from the analysis.

^a^Patients with a 6-month follow-up.

^b^Patients who were able to walk with or without a cane before injury with a 6-month follow-up, excluding any mortality case.

^c^Patients living at home before injury with a 6-month follow-up, excluding mortality cases.

### Association between patient characteristics and fall height

The results showed a negative correlation between fall height and the following pre-injury patient demographics: age at injury, proportion of women, not living at home, assisted walking, CCI, number of medications, mFI-5, and incidence of cervical ligament ossification (OPLL and DISH). Patients who fell from ground level tended to have a significantly higher prevalence of preexisting medical comorbidities, such as dementia, Parkinson’s disease, diabetes mellitus, cardiovascular disease, and osteoporosis; their mean medication number was > 4. The higher the fall height, the higher the incidence of cervical bony injuries, including vertebral fractures and/or dislocations; in contrast, the lower the fall height, the higher the incidence of cervical spinal cord injury without bony injuries. The neurological impairment (AIS) at the time of injury was not significantly associated with the fall height. The incidence of associated injuries was positively correlated with the fall height. There was no correlation between the surgical implementation rate and fall height (Table 3, Supplementary Table 2).

**Table 3:** Association between patient characteristics and fall height.

	Ground-level falls (n = 579)	Low falls (< 1 m) (n = 241)	High falls (≧1 m) (n = 333)	P-value for trend
**Pre-injury patient demographics**				
Mean age at injury (years)	77.5	76.5	73.9	< 0.001*
Sex: men, n (%)	384 (66.3)	147 (61.0)	251 (75.4)	0.01*
Mean body mass index (kg/m^2^)	22.0	21.9	22.4	0.17
Residence status: home, n (%)	534 (93.9)	235 (97.9)	324 (99.4)	< 0.001*
Walking capacity: walking w/ or w/o a cane, n (%)	522 (90.9)	230 (97.1)	332 (100.0)	< 0.001*
Mean Charlson comorbidity index	0.9	0.8	0.6	< 0.001*
Mean number of medications	4.7	4.1	3	< 0.001*
Mean modified 5-item frailty index	0.9	0.8	0.7	< 0.001*
**Presence of cervical ligamentous ossification**				
OPLL, n (%)	165 (28.5)	43 (17.8)	62 (18.7)	< 0.001*
OLF, n (%)	11 (1.9)	5 (2.1)	5 (1.5)	0.69
DISH, n (%)	104 (18.0)	26 (10.8)	26 (7.8)	< 0.001*
**Injury status**				
Cervical vertebral fracture, n (%)	238 (41.1)	139 (57.7)	218 (65.5)	< 0.001*
Cervical dislocation, n (%)	50 (8.6)	41 (17.0)	71 (21.3)	< 0.001*
Spinal cord injury, n (%)	453 (78.2)	158 (65.6)	224 (67.3)	< 0.001*
w/ bony injury, n (%)	127 (21.9)	68 (28.2)	126 (37.8)	< 0.001*
w/o bony injury, n (%)	326 (56.3)	90 (37.3)	98 (29.4)	< 0.001*
ASIA impairment scale				
A, n (%)	37 (6.4)	24 (10.0)	35 (10.6)	0.12
B, n (%)	28 (4.9)	15 (6.2)	14 (4.3)	
C, n (%)	145 (25.2)	48 (19.9)	73 (22.2)	
D, n (%)	240 (41.7)	71 (29.5)	98 (29.8)	
Associated injuries, n (%)	80 (13.8)	64 (26.6)	125 (37.5)	< 0.001*
**Treatment status**				
Surgery implementation, n (%)	334 (57.7)	137 (56.9)	205 (61.6)	0.27

Records with unknown or missing values were excluded from the analysis.

*OP﻿LL* Ossificationof the posterior longitudinal ligament, *OLF* Ossification of the ligamentum flavum, *DISH* Diffuse idiopathic skeletal hyperostosis, *ASIA* American Spinal Injury Association.

*Statistically significant trend between the levels of fall height in the Cochran–Armitage test or Jonckheere–Terpstra test.

### Association between outcomes at 6 months post-injury and fall height

For patients who were injured from ground-level falls, the mortality, walking independence, and home-discharge rates at 6 months post-injury were 9%, 67%, and 80%, respectively. Mortality was higher in patients who were injured at lower fall heights. Patients who fell from ground level tended to have higher complication rates, but there was no significant trend associated with the fall height. The independent walking rate was negatively correlated with the fall height. There was no significant difference in the home-discharge rates (Table 4).

**Table 4:** Association between outcomes at 6 months post-injury and fall height.

	Ground-level falls	Low falls (< 1 m)	High falls (≧1 m)	P-value for trend
**Mortality and complication rate**				
Number of subjects^a^	449	198	262	
Mortality, n (%)	40 (8.9)	18 (9.1)	12 (4.6)	0.049*
Complications, n (%)	176 (40.1)	64 (33.7)	88 (34.8)	0.13
Respiratory impairment, n (%)	31 (7.0)	13 (6.6)	17 (6.5)	0.79
Dysphagia, n (%)	35 (7.9)	12 (6.1)	18 (6.9)	0.65
Deep venous thrombosis, n (%)	5 (1.1)	5 (2.6)	4 (1.5)	0.57
Pulmonary embolism, n (%)	2 (0.5)	2 (1.0)	2 (0.8)	0.57
Pneumonia, n (%)	49 (11.0)	20 (10.2)	24 (9.2)	0.43
Urinary tract infection, n (%)	42 (9.5)	21 (10.7)	19 (7.3)	0.33
Cerebral infarction, n (%)	4 (0.9)	2 (1.0)	4 (1.5)	0.46
Myocardial infarction, n (%)	1 (0.2)	0 (0)	0 (0)	0.19
Delirium, n (%)	39 (8.8)	18 (9.1)	15 (5.7)	0.18
**Walking independence rate**				
Number of subjects^b^	371	175	249	
Walking capacity: walking w/ or w/o a cane, n (%)	248 (66.9)	131 (74.9)	193 (77.5)	0.03*
**Home-discharge rate**				
Number of subjects^c^	381	177	243	0.40
Returned to home, n (%)	306 (80.3)	142 (80.2)	202 (83.1)	
Admitted to a facility, n (%)	25 (6.6)	12 (6.8)	11 (4.5)	
In hospital, n (%)	50 (13.1)	23 (13.0)	30 (12.3)	

Records with unknown or missing values were excluded from the analysis.

^a^Patients with a 6-month follow-up.

^b^Patients who were able to walk with or without a cane before injury with a 6-month follow-up, excluding mortality cases.

^c^Patients living at home before injury with a 6-month follow-up, excluding mortality cases.

*Statistically significant trend between the levels of fall height in the Cochran–Armitage test or Jonckheere–Terpstra test.

### Prognostic factors for unfavorable outcomes in cases of ground-level falls

In patients of falls from ground level, the common factors for poor prognosis, including mortality, walking disability, and difficulty in home discharge, were older age and severe neurological impairment at the time of injury. As for the other independent associated factors, presence of bone injury was associated with mortality and walking disability, high CCI score was associated with mortality, and high mFI-5 was associated with walking disability and difficulty in home discharge (Table 5).

**Table 5:** Multivariate logistic regression analysis of the prognostic factors for unfavorable outcomes at 6 months post-injury in patients with ground-level falls.

Prognostic factors	OR	95% CIs	P-value
**Mortality**^a^			
Age at injury	1.13	1.07–1.20	< 0.001
Pre-injury Charlson comorbidity index	1.56	1.18–2.07	< 0.01
Presence of cervical bony injury	3.35	1.50–7.48	< 0.01
ASIA impairment scale	3.02	2.20–4.14	< 0.001
**Walking disability**^b^			
Age at injury	1.07	1.03–1.12	0.01
Pre-injury modified 5-item frailty index	1.63	1.11–2.39	0.01
Presence of cervical bony injury	2.03	1.11–3.74	0.02
ASIA impairment scale	4.79	3.23–7.10	< 0.001
**Difficulty in home discharge**^c^			
Age at injury	1.05	1.01–1.11	0.03
Pre-injury modified 5-item frailty index	1.91	1.28–2.85	< 0.01
ASIA impairment scale	3.72	2.60–5.31	< 0.001

All chi-squared tests for the models showed p < 0.001, and the Hosmer–Lemeshow tests showed p = 0.16, 0.84, and 0.81, respectively.

A correlation matrix was created to confirm that there was no high correlation between each explanatory variable.

*ASIA* American Spinal Injury Association, *CI* confidence interval, *OR* odds ratio.

^a^n = 449 with a 6-month follow-up.

^b^n = 371 who were able to walk with or without a cane before injury with a 6-month follow-up, excluding mortality cases.

^c^n = 381 who were living at home before injury with a 6-month follow-up, excluding mortality cases.

## Discussion

This is the first study to examine clinical characteristics including patients’ background, injury status, and prognosis of traumatic cervical spine injuries in older adults from a nationwide database according to the external cause of injury. In this study, three-quarters of cervical spine injuries in older adults were caused by falls, with ground-level falls accounting for the largest proportion. Furthermore, more than 50% of those aged 85 years and older were injured due to falling from ground level. When fall-induced cervical spine injuries were categorized and compared based on fall height, the patients’ backgrounds and injury statuses differed significantly. Patients injured from ground-level falls tended to be older, have poorer pre-injury health conditions (e.g., medical comorbidities and frailty), a higher rate of incomplete neurological impairment without cervical bony injuries, and less associated injuries compared with those who fell from higher heights. For ground-level falls, the mortality, walking independence, and home-discharge rates at 6 months post-injury were 9%, 67%, and 80%, respectively. The prognostic factors for unfavorable outcomes in patients injured due to ground-level falls were older age, pre-injury medical comorbidity and progression of frailty, presence of cervical bony injuries, and severity of neurological impairment at the time of injury according to AIS.

Falls are the most common causes of traumatic cervical spine injuries in older adults, accounting for 49–70% of geriatric cervical spine injuries^11,22,25,26^. Injuries from ground-level falls are particularly common in older adults, with more than 50% of traumatic cervical spine injuries caused by ground-level falls^9,27,28^. Our findings are consistent with those in previous reports, and the fact that the proportion of ground-level falls increased with age indicates that aging and cervical spine injury from minor trauma are clearly associated. Furthermore, cervical spine injuries caused by ground-level falls made it difficult for the patients to walk independently or be discharged from the hospital. Therefore, ground-level falls not only have a considerable impact on the health and quality of life of older people but also result in a social impact due to increased medical costs and need for nursing care^11,29^.

Several risk factors contribute to increased falls among older people and are generally classified as intrinsic or extrinsic factors. Intrinsic factors are individual-specific factors such as old age, chronic disease, muscle weakness, visual impairment, gait and balance disorders, cognitive impairment, and frailty. Extrinsic factors generally include medications (> 4 or psychoactive medication use), environmental and socioeconomic hazards, and hazardous activities^5,30–32^. Many of these factors are consistent with the characteristics of ground-level fall cases in this study. Furthermore, these factors interact with each other; thus, prevention of falls involves eliminating or modifying the risk factors whenever possible through education, optimal medical management, home care, and behavior modification^5^.

It is still uncertain why older people are prone to cervical spine injuries from minor traumatic falls from ground level. Degenerative changes, such as reduced flexion and extension mobility, spinal canal stenosis, and osteoporosis of the cervical spine in older people, increase the risk of cervical spinal injury after relatively minor trauma^33^. Moreover, the presence of ligamentous ossification diseases, such as OPLL and DISH, partly explains this phenomenon. Ligamentous ossification is common among Asian populations, and the prevalence of OPLL in patients with cervical spine injury is quite high. Additionally, most cervical spine injuries associated with OPLL are incomplete without bony injuries, and are caused predominantly by low-energy trauma^34,35^. In patients with DISH, minor trauma can cause spinal fractures, and OPLL has been reported to be significantly associated with neurological deterioration^36^. The prevalence of OPLL and DISH was also high in the present cohort, especially in patients who were injured during minor falls, which undoubtedly contributes to the occurrence of cervical spine injuries in older people.

Impacts to the head during a fall have also been considered as a causative factor in fall-induced cervical spine injuries in older adults. Compared to younger adults, older adults experienced more frequent impacts to the head during falls from ground level, and with greater acceleration^37^. Neck strength decreases by 35–45% between the ages of 20 and 60 years^38^. These age-related neuromuscular changes, resulting in an inability to control the head during a fall, are probably responsible for the greater proportion of impacts to the head in older adults^39,40^. Furthermore, medical care and life-prolonging treatments and medications increase the survival rate of older adults with poor health and functioning, which in turn leads to an increase in serious falls^10,41^. This concept is supported by the findings in the present study, since there were more cases of patients with preexisting medical comorbidities, medications, and advanced frailty in cases of ground-level falls compared to falls from a higher height.

In general, cervical spine injuries in older adults result in poor outcomes, with high rates of mortality and complications and a low rate of home discharge^42^. However, the mortality rate in recent years has been improving, and the rate in this study was similar to that reported in recent years^11,43,44^. In contrast to the increasing incidence of fall-induced cervical spine injuries, the mortality rate has been decreasing, probably due to advanced medical management of these patients^43,45^. However, the prognosis of cervical spine injuries in older adults remains unfavorable. Although an association between the severity of neurological injury and mortality has been reported^23^, this appears to be a contradictory finding, as older adults tend to have higher rates of incomplete injury caused by low falls. It has been reported that the severity of neurological impairment at the time of injury alone is not sufficient to determine the prognosis in older adults, and this apparent contradiction is best explained by the presence of confounding factors that affect mortality^46^. The presence of comorbidities has been shown to be a risk factor for mortality in patients with spinal cord injury, and the CCI was found to be a more reliable predictor of in-hospital mortality than age or the severity of neurological impairment^20^. Additionally, decreased physiological reserve, widely known as "frailty", has been demonstrated to be associated with poor outcomes related to spinal cord injury, such as adverse events, mortality, and length of hospital stay; however, this association in older adults remains controversial^21,47^. This study indicates that the progression of frailty, a unique feature in ground-level fall cases, is significantly related to unfavorable prognoses such as walking disability and difficulty in home discharge. Functional recovery after spinal cord injury in older adults was considered to be worse due to decreased physiological reserve, even though neurological improvement was comparable to that in younger patients^48,49^. Thus, the progression of frailty seems to lead to a negative chain of events that not only increases the incidence of cervical spine injury due to falls, but also worsens its prognosis.

Finally, several factors prior to a fall-induced cervical spine injury, such as aging, underlying comorbidities, and frailty, significantly affect the onset of injury and its outcomes. In addition, falling as a cause of injury has been reported to be one of the significant predictors of long-term mortality after trauma^50^. The findings of this study support this, with a particularly poor prognosis in the case of ground-level falls, which were associated with pre-injury factors. Thus, ground-level falls should be treated separately from other injuries, as ground-level falls can be an indicator of pre-injury poor health and have a higher risk of unfavorable outcomes. It is therefore essential to improve the general health condition of older adults as well as to implement multifaceted fall prevention programs. Both the appropriate management of underlying comorbidities and frailty would decrease not only the incidence but also the severity of cervical spine injury in older adults. In Japan, a frailty questionnaire was introduced in general health examinations in 2020, and further accumulation of data and countermeasures based on it are expected.

The strength of the present study was that it was conducted using a nationwide, multicenter investigation to examine cervical spine injuries in older adults by the external cause of injury. However, there are several limitations, including the study’s retrospective design using a heterogeneous cohort without a control group and that it was not an exhaustive survey. Furthermore, it was difficult to perfectly compare the treatment outcomes by external causes of injury since there was no standardized treatment approach, including surgical indications and rehabilitation methods, and the patients’ backgrounds and injury statuses differed significantly depending on the external causes of injury. Moreover, the survey was conducted in Japan, which has a unique background with the world's most advanced aging population. However, the entire world is moving toward a super-aging society, and cervical spine injury in older adults has become an urgent issue. We believe that the results of this study will provide valuable information as a model case for the aging society.

## Conclusion

Falls were the most common cause of traumatic cervical spine injury in older adults, with ground-level falls accounting for the largest proportion. When falls were categorized and compared based on the fall height, it was found that the patients’ backgrounds and injury statuses differed markedly. Furthermore, patients injured from ground-level falls tended to have poorer pre-injury health conditions, such as medical comorbidities and frailty, compared with those who fell from higher heights. For ground-level falls, mortality, walking independence, and home-discharge rates at 6 months post-injury were 9%, 67%, and 80%, respectively, with a high CCI associated with higher mortality and a high mFI-5 associated with walking disability and difficulties during home discharge, independent of age or the severity of neurological impairment at the time of injury. Thus, it is important to treat and manage older patients who sustain cervical spine injuries due to minor trauma, such as a ground-level fall, considering that they may have relatively poor pre-injury health conditions, making them prone to unfavorable outcomes.

## Supplementary Information

Supplementary Tables.

## Data Availability

The datasets used and analyzed during the current study are available from the corresponding author on reasonable request.
