# Supplementary material for: Differences in clinical characteristics of cervical spine injuries in older adults by external causes: a multicenter study of 1512 cases
Source: Sci Rep. 2022 Sep 23;12:15867. doi: 10.1038/s41598-022-19789-y (PMC9508126; doi:10.1038/s41598-022-19789-y)
Supplement: Supplementary file 1 — Supplementary Tables. [file 41598_2022_19789_MOESM1_ESM.docx]

**Supplementary tables**

**Supplementary Table 1. Patient characteristics by the external cause of injury**

|  | Overall  (n=1,512) | Falls  (n=1,153) | Traffic accidents  (n=287) | Others  (Including unspecified)  (n=72) |
| --- | --- | --- | --- | --- |
| ●Pre-injury patient demographics |  |  |  |  |
| Medical comorbidity |  |  |  |  |
| Cerebrovascular disease, n (%) | 145 (9.9) | 115 (10.3) | 24 (8.9) | 6 (8.5) |
| Dementia, n (%) | 95 (6.5) | 84 (7.5) | 10 (3.7) | 1 (1.4) |
| Parkinson’s disease, n (%) | 22 (1.5) | 21 (1.9) | 1 (0.4) | 0 (0) |
| Hypertension, n (%) | 731 (49.6) | 568 (50.3) | 133 (48.5) | 30 (42.3) |
| Diabetes mellitus, n (%) | 330 (22.6) | 270 (24.1) | 39 (14.6) | 21 (29.6) |
| Cardiovascular disease, n (%) | 227 (15.6) | 191 (17.1) | 28 (10.5) | 8 (11.3) |
| Respiratory disease, n (%) | 81 (5.6) | 66 (5.9) | 12 (4.5) | 3 (4.2) |
| Renal disease, n (%) | 73 (5.0) | 57 (5.1) | 9 (3.4) | 7 (9.9) |
| Musculoskeletal disorders, n (%) | 190 (13.0) | 152 (13.6) | 33 (12.2) | 5 (7.0) |
| Osteoporosis, n (%) | 99 (6.8) | 81 (7.3) | 16 (6.0) | 2 (2.9) |
| Blood test data at admission |  |  |  |  |
| Mean serum total protein value (g/dL) | 6.6 | 6.6 | 6.6 | 6.8 |
| Mean serum hemoglobin value (g/dL) | 12.7 | 12.6 | 12.6 | 13.3 |
| ●Injury status |  |  |  |  |
| Associated injuries |  |  |  |  |
| Head injury, n (%) | 217 (14.4) | 144 (12.5) | 68 (23.9) | 5 (6.9) |
| Thoracic injury, n (%) | 88 (5.9) | 39 (3.4) | 48 (17.0) | 1 (1.4) |
| Abdominal injury, n (%) | 22 (1.5) | 8 (0.7) | 14 (4.9) | 0 (0) |
| Upper limb injury, n (%) | 77 (5.1) | 48 (4.1) | 27 (9.5) | 2 (2.8) |
| Lower limb injury, n (%) | 55 (3.7) | 24 (2.1) | 31 (10.9) | 0 (0) |
| Pelvic fracture, n (%) | 27 (1.8) | 14 (1.2) | 12 (4.2) | 1 (1.4) |
| Thoracolumbar vertebral fracture, n (%) | 91 (6.0) | 70 (6.1) | 18 (6.3) | 3 (4.2) |
| Records with unknown or missing values were excluded from the analysis. | | | | |

**Supplementary Table 2. Association between patient characteristics and fall height**

|  | Ground-level falls  (n=579) | Low falls  (<1 m)  (n=241) | High falls  (≧1 m)  (n=333) | P-value for trend |
| --- | --- | --- | --- | --- |
| ●Pre-injury patient demographics |  |  |  |  |
| Medical comorbidity |  |  |  |  |
| Cerebrovascular disease, n (%) | 65 (11.6) | 25 (10.6) | 25 (7.7) | 0.07 |
| Dementia, n (%) | 54 (9.6) | 19 (8.0) | 11 (3.4) | <0.001* |
| Parkinson’s disease, n (%) | 17 (3.1) | 3 (1.3) | 1 (0.3) | <0.01* |
| Hypertension, n (%) | 299 (52.6) | 123 (51.9) | 146 (45.2) | 0.05 |
| Diabetes mellitus, n (%) | 149 (26.6) | 56 (23.6) | 65 (20.3) | 0.03* |
| Cardiovascular disease, n (%) | 114 (20.4) | 38 (16.0) | 39 (12.2) | <0.01* |
| Respiratory disease, n (%) | 27 (4.9) | 17 (7.2) | 22 (6.9) | 0.19 |
| Renal disease, n (%) | 31 (5.6) | 11 (4.6) | 15 (4.7) | 0.53 |
| Musculoskeletal disorders, n (%) | 84 (15.0) | 35 (14.8) | 33 (10.3) | 0.06 |
| Osteoporosis, n (%) | 46 (8.2) | 22 (9.3) | 13 (4.1) | 0.04* |
| Blood test data at admission |  |  |  |  |
| Mean serum total protein value (g/dL) | 6.7 | 6.6 | 6.6 | 0.11 |
| Mean serum hemoglobin value (g/dL) | 12.6 | 12.5 | 12.8 | 0.14 |
| ●Injury status |  |  |  |  |
| Associated injuries, n (%) |  |  |  |  |
| Head injury, n (%) | 51 (8.8) | 34 (14.1) | 59 (17.8) | <0.001* |
| Thoracic injury, n (%) | 5 (0.9) | 6 (2.5) | 28 (8.5) | <0.001* |
| Abdominal injury, n (%) | 3 (0.5) | 0 (0) | 5 (1.5) | 0.13 |
| Upper limb injury, n (%) | 9 (1.6) | 11 (4.6) | 28 (8.4) | <0.001* |
| Lower limb injury, n (%) | 4 (0.7) | 4 (1.7) | 16 (4.8) | <0.001* |
| Pelvic fracture, n (%) | 2 (0.4) | 3 (1.2) | 9 (2.7) | <0.01* |
| Thoracolumbar vertebral fracture, n (%) | 14 (2.4) | 15 (6.2) | 41 (12.4) | <0.001* |
| Records with unknown or missing values were excluded from the analysis.  ^*^Statistically significant trend between the levels of fall height in the Cochran–Armitage test or Jonckheere–Terpstra test | | | | |

1
